# Birmingham Behçet’s service: classification of disease and application of the 2014 International Criteria for Behçet’s Disease (ICBD) to a UK cohort

**DOI:** 10.1186/s12891-017-1463-y

**Published:** 2017-03-11

**Authors:** Tim Blake, Luke Pickup, David Carruthers, Erika Marie Damato, Alastair Denniston, John Hamburger, Claire Maxton, Debbie Mitton, Philip I. Murray, Peter Nightingale, Ana Poveda-Gallego, Andrea Richards, Andrew Whallett, Deva Situnayake

**Affiliations:** 1 Rheumatology Department, Birmingham City Hospital, Sandwell and West Birmingham Hospitals NHS Trust, Dudley Rd, Birmingham, B18 7QH UK; 2 Institute of Inflammation and Ageing, University of Birmingham College of Medical and Dental Sciences, Birmingham, UK; 3 Rheumatology, Sandwell and West Birmingham Hospitals NHS Trust, Birmingham, UK; 4 Ophthalmology, Birmingham and Midland Eye Centre, Birmingham, UK; 5 Oral Medicine, Sandwell and West Birmingham Hospitals NHS Trust, Birmingham, UK; 6 Wolfson Computer Laboratory, University Hospital Birmingham NHS Foundation Trust, Birmingham, UK; 7 School of Dentistry, University of Birmingham, Birmingham, UK; 8 Oral Medicine, Birmingham Community Healthcare NHS Trust, Birmingham, UK; 9 Rheumatology, Dudley Group of Hospitals NHS Foundation Trust, Birmingham, UK

**Keywords:** Behçet’s disease, Epidemiology, History of medicine, Inflammation, Auto-inflammatory disorders

## Abstract

**Background:**

This study reports on the analysis of the application and diagnostic predictability of the revised 2014 ICBD criteria in an unselected cohort of UK patients, and the ensuing organ associations and patterns of disease.

**Methods:**

A retrospective cohort study was conducted using a database of electronic medical records. Three categories were recognised: clinically defined BD, incomplete BD and rejected diagnoses of BD. We applied the ISG 1990 and ICBD 2014 classification criteria to these subgroups to validate diagnostic accuracy against the multidisciplinary assessment.

**Results:**

Between 2012 and 2015, 281 patients underwent initial assessment at an urban tertiary care centre: 190 patients with a confirmed diagnosis of BD, 7 with an incomplete diagnosis, and 84 with a rejected diagnosis. ICBD 2014 demonstrated an estimated sensitivity of 97.89% (95% CI: 94.70 to 99.42) and positive likelihood ratio of 1.21 (1.10 to 1.28). The strongest independent predictors were: Central nervous lesions (OR = 10.57, 95% CI: 1.34 to 83.30); Genital ulceration (OR = 9.05, 95% CI: 3.35 to 24.47); Erythema nodosum (OR = 6.59, 95% CI: 2.35 to 18.51); Retinal vasculitis (OR = 6.25, 95% CI: 1.47 to 26.60); Anterior uveitis (OR = 6.16, 95% CI: 2.37 to 16.02); Posterior uveitis (OR = 4.82, 95% CI: 1.25 to 18.59).

**Conclusions:**

The ICBD 2014 criteria were more sensitive at picking up cases than ISG 1990 using the multidisciplinary assessment as the gold standard. ICBD may over-diagnose BD in a UK population. Patients who have an incomplete form of BD represent a distinct group that should not be given an early diagnostic label. Behçet’s disease is a complex disease that is best diagnosed by multidisciplinary clinical assessment. Patients in the UK differ in their clinical presentation and genetic susceptibility from the original descriptions. This study also highlights an incomplete group of Behçet’s patients that are less well defined by their clinical presentation.

## Background

Behçet’s disease (BD) is a complex multisystem auto-inflammatory disorder, which in its classic form, presents with recurrent oral aphthous ulcers, genital ulcers, and uveitis. Its aetiology is unknown but likely involves interplay between genetic and environmental factors [1]. BD has a very heterogeneous and unpredictable phenotype with the potential to involve the cardiovascular, renal, gastrointestinal, pulmonary, vascular, musculoskeletal, urological and central nervous systems to varying degrees [2–4].

Behçet’s disease is more common, and often more severe, along the ancient Silk Road, which extended from eastern Asia to the Mediterranean [5, 6]. It is most common in Turkey (80 to 370 cases per 100,000) [6], while the prevalence is much lower in Northern European and North American populations (1 per 15,000 to 1 per 500,000) [7]. The first symptoms often occur in young adults between 20 and 40 years of age but it is also infrequently seen in children. Familial clustering has been reported, although most cases of Behçet’s are thought to be sporadic in onset [8, 9].

As there is no universally accepted pathognomonic test for BD, diagnosis is based on the recognition of a particular but variable group of clinical manifestations. In 1990, these features were incorporated into the International Study Group (ISG) diagnostic/classification criteria based on data from a computer analysis of 914 patients with BD and 308 diseased controls with clinical features mimicking those of BD [10]. Although these were originally intended for the definition of patients participating in research programmes, they have since been shown to perform well in a clinical context and may be helpful in establishing a diagnosis [11]. More recent evaluations of the ISG collaboration have found lower sensitivity when compared to other classification criteria, leading to an initiative to develop newer criteria. In 2014, an international team from 27 countries (not including the UK) described the new International Criteria for Behçet’s disease (ICBD), capable of “performing with good discriminatory potential regardless of country” and being “intuitive and easy to use in a wide variety of settings”. In comparison to the earlier ISG criteria, the ICBD 2014 criteria included both vascular and neurological features, and assigned more points for the presence of oral or genital aphthosis and ocular findings. In the published evaluation, the newly proposed criteria exhibited much improved sensitivity over the older widely accepted ISG criteria while maintaining specificity. As a result, it was proposed that these revised criteria could be used as a tool for mass screening and identification of possible Behçet’s patients in different clinical settings. The authors attempted to standardise and define a group of patients who had definite disease, and contrast these with patients who had fewer clinical manifestations and were unlikely to have Behçet’s [12].

Here we report on the application and performance of both the ISG and ICBD criteria to a cohort of unselected patients referred to the Birmingham Behçet’s Syndrome National Centre of Excellence since its inception in July 2012 to July 2015. The clinic was established in 1990 and has traditionally adopted a multidisciplinary approach to the management of patients with BD [13]. The aim of the study was to determine the frequency distribution of clinical characteristics for patients with clinically confirmed BD, possible but unlikely (termed ‘incomplete’) BD and a rejected diagnosis of BD. We compared the ISG and ICBD points-based scoring criteria with the gold standard multidisciplinary clinical assessment process (a core feature of our service design) to determine the probability of BD in our UK-based cohort, and reviewed the potential impact of the application of the new classification system as a screening tool for new referrals to the service. We investigated how many patients, who presented with typical clinical features of BD, would be reclassified if the newer ICBD 2014 classification criteria were employed.

## Methods

This was a retrospective study of an unselected inception cohort in a tertiary referral centre. It was conducted in accordance with the Declaration of Helsinki, and approved by the local Trust Research and Development team and The London-Westminster Research Ethics Committee. Informed consent, including that from a responsible legal guardian in certain instances, was obtained from all patients before their details were entered onto the electronic database.

### Data collection

All patients seen at the Birmingham Centre of Excellence for Behçet’s disease since the Centre’s inception in July 2012 until July 2015 were recruited consecutively. Data were obtained from the in-house database for all 281 patients irrespective of disease duration. Patients’ clinical and demographic characteristics were collected and summarised, so as to discern local patterns of disease. With respect to pathergy, we assigned a total score based on the result of any formal testing as well as reported reaction, as this phenomenon is not currently routinely assessed in a UK population.

Patient diagnosis was established at the time of first presentation following multidisciplinary combined clinical assessment. Clinicians involved in this assessment included specialists in Rheumatology, Ophthalmology, Oral Medicine, Gynaecology, and where appropriate Gastroenterology and Dermatology, after review of the referral information and pre-clinic contact with the patient. Diagnoses were made collaboratively following independent specialist assessment. Three categories were recognised: clinically defined BD, incomplete BD and rejected diagnoses of BD. The groups were analysed separately to determine clinically defining or discriminating features. Patients who were classified as incomplete BD were felt to represent an interesting category, demonstrating some features consistent with but not necessarily diagnostic of BD, and not needing systemic disease modifying therapies at the time of assessment. Patients who presented with some clinical features of BD but were believed to have an alternative diagnosis were classified as a rejected group and usually discharged from follow up. We applied the ISG 1990 and ICBD 2014 classification criteria to these subgroups to validate diagnostic accuracy against the gold standard multi-disciplinary assessment process in our UK based cohort. A selection of cases were subsequently analysed in more detail: those who were clinically diagnosed following multidisciplinary review but nevertheless went on to meet the newer ICBD 2014 criteria. We compared variables between those patients who were newly classified as BD by ICBD with those in the BD group who were not reclassified.

### Statistical analysis

All statistical analyses were conducted using IBM SPSS Statistics for Windows, Version 22.0 (Armonk, NY: IBM Corp.). Levels of continuous variables were expressed as means ± standard deviations. Continuous variables were compared between the categories of disease using a one-way analysis of variance with Tukey’s Honestly Significant Difference test, and categorical variables were compared with Fisher’s exact test. Diagnostic accuracies of ISG 1990 and ICBD 2014 criteria were represented by sensitivities, specificities, and likelihood ratios, where patients in the incomplete group were excluded from analysis. Odds ratios were obtained from a multivariable logistic regression analysis among variables with *P* values ≤ 0.05 in univariable analyses. For all statistical evaluations, *P* values ≤ 0.05 were considered to indicate statistical significance.

## Results

A total of 281 patients were analysed: 190 patients with a confirmed diagnosis of BD, 7 with an incomplete diagnosis, and 84 with a rejected diagnosis following multispecialty clinical evaluation. Table 1 displays demographic data for the patient groups.

**Table 1:** Patient characteristics according to clinical diagnosis

	Overall (*n* = 281)	BD (*n* = 190)	Incomplete BD (*n* = 7)	Rejected diagnosis (*n* = 84)
Age (years, mean +/− SD)	41.27 ± 13.63	43.42 ± 12.08	34.43 ± 7.50	36.99 ± 16.04
Age category (%)				
< 14 14–18 19–25 26–30 31–40 41–50 51–60 61–70 71–80 81–90	1 (0.4) 10 (3.6) 23 (8.2) 28 (10.0) 79 (28.1) 70 (24.9) 47 (16.7) 18 (6.4) 4 (1.4) 1 (0.4)	0 (0) 2 (0.7) 9 (3.2) 20 (7.1) 55 (19.6) 48 (17.1) 40 (14.2) 13 (4.6) 3 (1.1) 0 (0)	0 (0) 0 (0) 2 (0.7) 0 (0) 3 (1.1) 2 (0.7) 0 (0) 0 (0) 0 (0) 0 (0)	3 (1.1) 8 (2.9) 12 (4.3) 8 (2.9) 19 (6.8) 20 (7.1) 7 (2.5) 5 (1.8) 1 (0.4) 1 (0.4)
Sex, male (*N* (%))	98 (34.9)	72 (25.6)	3 (1.1)	23 (8.2)
Ethnicity (*N* (%))				
Arab Asian – Indian subcontinent Black African Black Caribbean Chinese Greek Italian Maori White/Black Caribbean White British Unspecified	4 (1.4) 21 (7.5) 2 (1.1) 2 (0.7) 8 (2.9) 1 (0.4) 1 (0.4) 1 (0.4) 172 (61.2) 66 (23.5) 2 (0.7)	4 (1.42) 20 (7.1) 2 (1.1) 2 (0.7) 7 (2.5) 0 (0) 1 (0.4) 1 (0.4) 123 (43.8) 27 (9.6) 2 (0.7)	0 (0) 0 (0) 0 (0) 0 (0) 0 (0) 0 (0) 0 (0) 0 (0) 5 (1.8) 2 (0.7) 0 (0)	0 (0) 1 (0.4) 0 (0) 0 (0) 1 (0.4) 1 (0.4) 0 (0) 0 (0) 44 (15.7) 37 (13.2) 0 (0)

One-way analysis of variance indicated that the mean age of the three groups was significantly different. Post-hoc Tukey’s Honestly Significant Difference test also showed that the mean age was significantly different between the BD and rejected groups (*P* < 0.001). The proportion of males was not significantly different between the groups (*P* = 0.205, Fisher’s exact test), however for ethnicity the three groups differed significantly (*P* = 0.002, Fisher’s exact test).

Forty-two of the 281 cases failed to meet ISG 1990 criteria; 38 of these went on to meet the newer ICBD 2014 criteria (reclassified). Twenty-six cases were classified as BD by the ISG criteria but were not confirmed clinically, all of whom were in the rejected diagnosis group; this increased to 68 cases for the ICBD 2014 criteria. The 16 patients who were both clinically and ICBD negative were given alternative diagnoses based on clinical assessment. These included ocular toxoplasmosis, vesicobullous autoimmune diseases, inflammatory bowel disease, Sweet’s syndrome, idiopathic aphthous ulceration, nutritional and dietary deficiencies, and potential auto-inflammatory syndromes. All of the seven patients who were categorised as having incomplete BD exhibited oral ulcers, along with various other clinical manifestations that included: acneiform rash, pseudofolliculitis, non-specific arthralgia, inflammatory arthritis, enthesopathy, fibromyalgia, chronic diarrhoea and peripheral neuropathy. In addition, none of these patients satisfied either the ISG 1990 or ICBD 2014 criteria.

A comparison of the ISG 1990 and ICBD 2014 criteria and subsequent diagnostic accuracies compared with the gold standard clinical diagnoses are displayed in Table 2.

**Table 2:** Diagnostic accuracy of ISG 1990 and ICBD 2014 criteria in Birmingham cohort

	ISG (95% CI)	ICBD (95% CI)
Sensitivity	77.9% (71.3 to 83.6)	97.9% (94.7 to 99.4)
Specificity	69.1% (58.0 to 78.7)	19.1% (11.3 to 29.1)
Positive Likelihood ratio	2.52 (1.85 to 3.53)	1.21 (1.10 to 1.28)
Negative Likelihood ratio	0.32 (0.24 to 0.43)	0.11 (0.03 to 0.34)
Disease prevalence	69.3% (63.5 to 74.8)	69.3% (63.5 to 74.8)
Positive Predictive Value	85.1% (78.9 to 90.0)	73.2 (67.3 to 78.6)
Negative Predictive Value	58.0 (47.7 to 67.8)	80.0 (56.3 to 94.3)

**Fig. 1:**
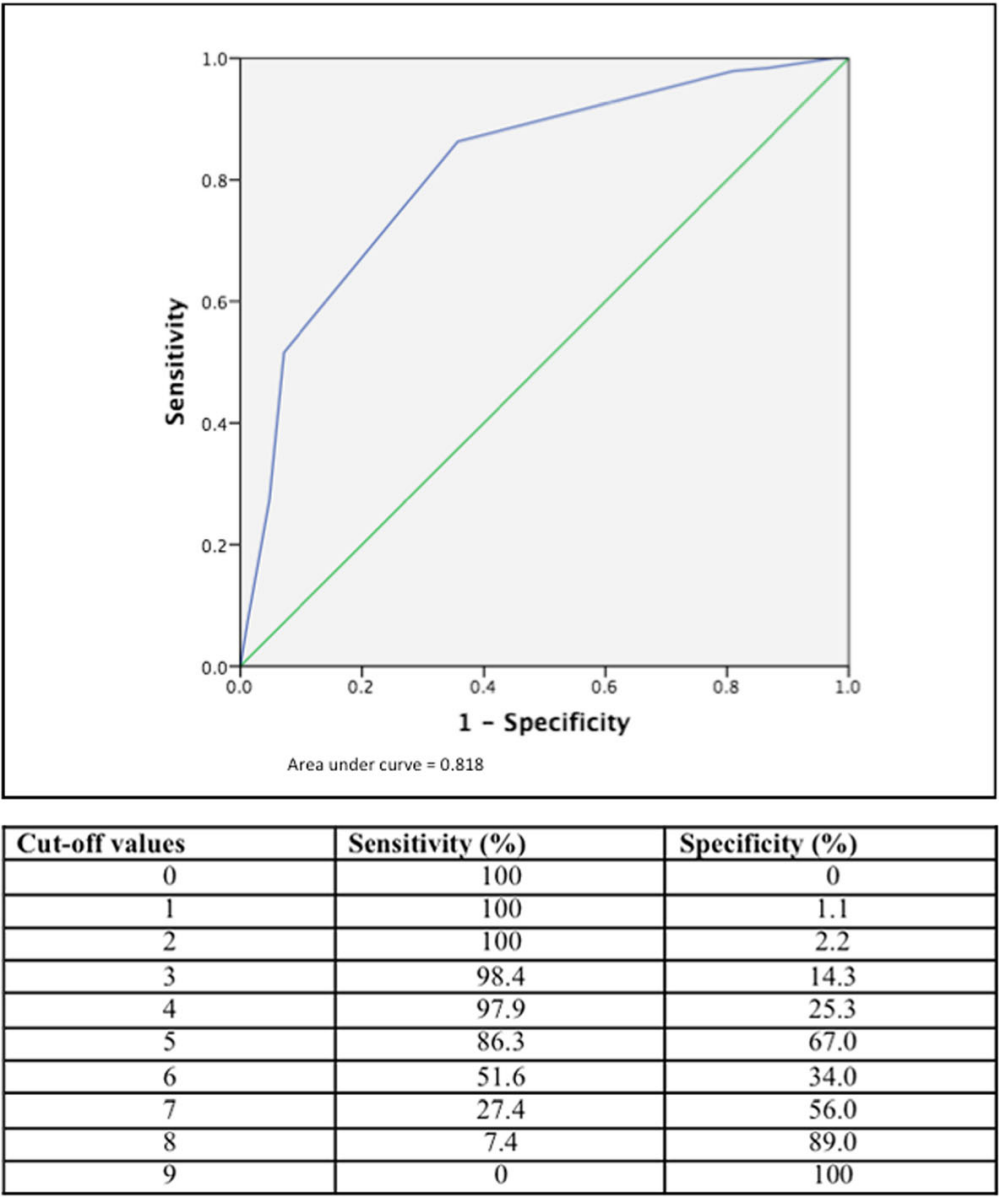
The receiver operator curve and sensitivity and specificity for the ICBD criteria

The Receiver Operating Characteristic (ROC) curve was calculated for the 2014 criteria in the study population (0–9 cut-off points; *n* = 274) (Fig. 1). The area under the ROC curve (AUC) was 0.818.

The frequencies of individual clinical features according to diagnosis are shown in Table 3. The majority of patients referred to the Behçet’s service had recurrent oro-genital aphthous ulceration, however three of the patients with confirmed BD did not present with oral ulcers; all of these had significant inflammatory eye disease that was typical of BD.

**Table 3:** Frequencies of individual clinical features according to clinical diagnosis

	BD (*n* = 190) (%)	Incomplete BD (*n* = 7) (%)	Rejected diagnosis (*n* = 84) (%)	*P*-value^a^
Oral aphthosis	187 (98.4)	7 (100.0)	80 (95.2)	0.206
Genital aphthosis	161 (84.7)	0 (0)	58 (69.0)	0.005
Anterior uveitis	83 (43.7)	0 (0)	12 (14.3)	<0.001
Posterior uveitis	54 (28.4)	0 (0)	3 (3.6)	<0.001
Erythema nodosum	49 (25.8)	0 (0)	5 (6.0)	<0.001
Acneiform lesions	42 (22.1)	2 (28.6)	16 (19.0)	0.632
Pseudofolliculitis	41 (21.6)	1 (14.3)	10 (11.9)	0.065
Retinal vasculitis	33 (17.4)	0 (0)	4 (4.8)	0.004
Pathergy test positivity	23 (12.1)	0 (0)	7 (7.1)	0.408
Large vein thrombosis	21 (11.1)	1 (14.3)	4 (4.8)	0.114
Central nervous lesion	18 (9.5)	0 (0)	1 (1.2)	0.010
Arterial thrombosis	8 (4.2)	1 (14.3)	1 (1.2)	0.283
Skin aphthosis	7 (3.7)	0 (0)	0 (0)	0.104
Phlebitis	5 (2.6)	0 (0)	0 (0)	0.328
Superficial thrombophlebitis	5 (2.6)	0 (0)	0 (0)	0.328
Peripheral nervous lesion	3 (1.6)	1 (14.3)	1 (1.2)	1.000

^a^Using Fisher’s exact test

All the statistically significant variables above remained significant when entered together into a multivariable binary logistic regression model:

Genital ulceration *P* < 0.001: Odds ratio 9.05 with 95% confidence interval 3.35 to 24.47; Anterior uveitis *P* < 0.001: Odds ratio 6.16 with 95% confidence interval 2.37 to 16.02; Posterior uveitis *P* = 0.022: Odds ratio 4.82 with 95% confidence interval 1.25 to 18.59; Erythema nodosum *P* < 0.001: Odds ratio 6.59 with 95% confidence interval 2.35 to 18.51; Retinal vasculitis *P* = 0.013: Odds ratio 6.25 with 95% confidence interval 1.47 to 26.60; Central nervous lesions *P* = 0.025: Odds ratio 10.57 with 95% confidence interval 1.34 to 83.30.

Thirty-eight patients were given a clinical diagnosis of BD based on multidisciplinary clinical assessment and met the ICBD but not the earlier ISG criteria. Table 4 represents a comparison between those patients in the BD group who were reclassified based on ICBD and those who were not. The reclassified patients exhibited higher vascular and neurological scores.

**Table 4:** Comparison of variables between patients who were newly classified as Behçet’s disease according to ICBD classification criteria

Variables	Patients reclassified as BD (*n* = 38)	Patients not reclassified as BD (*n* = 152)	*P*-value^a^
Characteristics			
Age (years) Sex, male (*n* %))	45.0 ± 13.9 16 (42.1)	40.8 ± 13.5 56 (36.8)	
Clinical manifestations (n (%)			
Oral aphthosis Genital aphthosis Pseudofolliculitis Acneiform rash Erythema nodosum Skin aphthosis Anterior uveitis Posterior uveitis Retinal vasculitis Peripheral nervous Central nervous Arterial thrombosis Large vein thrombosis Phlebitis Superficial phlebitis Pathergy test positivity	38 (100) 25 (65.8) 0 (0) 0 (0) 0 (0) 0 (0) 11 (28.9) 8 (21.1) 5 (13.2) 1 (2.6) 10 (26.3) (7.9) (10.5) 1 (2.6) 1 (2.6) 0 (0)	149 (98.0) 136 (89.5) 41 (27.0) 42 (27.6) 49 (32.2) 7 (4.6) 72 (47.4) 46 (30.3) 28 (18.4) 2 (1.3) 8 (5.3) 5 (3.3) 17 (11.2) 4 (2.6) 4 (2.6) 23 (15.1)	1.000 <0.001 <0.001 <0.001 <0.001 0.348 0.045 0.318 0.632 0.490 <0.001 0.199 1.000 1.000 1.000 0.005

^a^Using Fisher’s exact test

## Discussion

Our data shares some similarities with that obtained from the development of the new multinational classification scheme for BD [12]. In our cohort, ICBD 2014 demonstrated an estimated sensitivity of 97.89% (95% CI: 94.70 to 99.42), compared to 94.8% (95% CI: 93.40 to 95.9) quoted in the original validation set, considerably higher than that of the ISG 1990 criteria (77.89%). We found a positive likelihood ratio of 1.21 (1.10 to 1.28). Nevertheless, we measured the specificity of ICBD 2014 to be much lower than that revealed in the original data set (19.05% (11.30 to 29.08), compared with 90.5% (95% CI: 87.9 to 92.8%). The reason for this discrepancy is that, in the new classification, a score of four can be achieved solely with the presence of oro-genital ulcers; however following clinical assessment patients were often diagnosed with an alternative explanation for their ulcers, such as idiopathic or post-infectious aetiology, which would have given rise to higher false positive rates. This is supported by the finding that the symptoms showing the highest frequency but the least discriminatory utility in our cohort were oral and genital ulcers.

There were three patients who were diagnosed with BD on clinical grounds but did not report classic recurrent oral aphthosis; all of these were diagnosed following identification of characteristic ophthalmic changes of panuveitis and retinal vasculitis. These patients would not have satisfied the earlier ISG 1990 criteria for diagnosing BD given the lack of recurrent oral ulcers; however it is recognised that ocular disease may be the initial manifestation in about 20% of cases. In one series, anterior uveitis was present in 59% of cases, posterior uveitis was present in 76% of cases, and panuveitis was present in 88.1% of cases [14]. Since there is no pathognomonic clinical sign or laboratory test to distinguish BD from other uveitic causes, the diagnosis must be made based on characteristic ocular and systemic findings in the absence of evidence of other disease that can explain the findings. This has led some to develop diagnostic or classification criteria, for use in the uveitis community, that rely on a minimum number and/or combination of clinical findings to identify Behçet’s disease [15].

The incomplete group, described in the original combined testing and validation sets of the ICBD criteria as those with ‘possible but unlikely BD’, demonstrated undisputed presence of oral ulcers (100%), along with several skin and thrombotic manifestations. Patients in this group were predominantly female. The authors believe that these patients form an interesting subgroup, which should ideally be monitored for the development of more specific target organ associations but should not be given a diagnostic label due to uncertainty about progression and treatment. None of the patients in the incomplete group were started on systemic immunomodulatory therapies. In the reclassification group, more patients were female, and central nervous system lesions appeared statistically predictive for the development of BD. Peripheral nervous system lesions also showed a trend towards redefining disease according to the newer criteria. There was no history of pathergy nor was it observed on testing in all patients in this group.

Behçet’s disease, like most other rheumatic diseases, lacks a gold-standard test with a high degree of sensitivity and specificity, making it necessary to develop classification and diagnostic criteria to guide researchers and clinicians. Classification criteria are designed to define a homogenous population with similar clinical features suitable for research studies. They are essential for our understanding of disease pathogenesis, treatments outcomes, for entry into clinical trials, and as such, increase the specificity for underlying disease while at the same time lose sensitivity on Receiver Operating Characteristic (ROC) curve analysis. Conversely, the goal of diagnostic criteria is to have a high sensitivity and positive predictive value (PPV) so as not to exclude individuals with possible disease. The specificity and PPV of ICBD was considerably lower than ISG in this cohort, which reflects the degree of false positive cases referred to the service and falsely thought to have BD. These indices are likely to be even more truncated in local Rheumatology centres where the prevalence of true BD is far lower. For these reasons, it is important for criteria to be tailored to the practice setting and clinicians to formulate a diagnosis based on sound clinical judgement and experience in rare conditions.

To further understand how “universal” classification criteria can be employed in individual clinical settings, one should appreciate the role of Bayes’ theorem, named after the 18^th^ century English statistician, philosopher and minister. It states that the odds of having a disease is equal to the pre-test odds multiplied by the likelihood ratio; the former being determined by the prevalence in the population and the latter by the sensitivity and specificity in the data set [16]. Both types of criteria are highly dependent on the disease prevalence in the patient population being investigated. Bayes informs us that a set of criteria can only be accurately applied to the cohort for which it was designed. In light of this, it is important to realise that there are important genotypic and phenotypic variations in disease expression between different populations in BD.

The genetic locus most widely studied in BD is the human leukocyte antigen (HLA) complex on chromosome 6p21. Disease susceptibility has consistently been associated with polymorphisms in the HLA-B gene, particularly HLA-B*51 [17]. A recent meta-analysis showed a significant increase in the risk of HLA-B*51 carriers to develop BD compared with non-carriers across multiple geographic locations [18]. Nevertheless, the HLA-B*51 association is not invariable: the relative risk of disease with this haplotype is known to be stronger in Turkish, Middle Eastern, and Japanese populations than in Caucasians [19], and ethnic differences are thought to have a major impact on clinical expression of BD [20]. More recently, Caucasian patients from the UK have also been found to express HLA-B*57, another susceptibility gene that carries a relative risk of disease equivalent to that of HLA-B*51 [21]. Other HLA alleles may also increase or decrease the risk for Behçet’s in various populations and in men and women [22–25]. Moreover, in a UK population, pathergy reaction is a relatively rare phenomenon when tested for [26]. There are likely to be further gender-specific differences such as those identified by the German Adamantiades Behcet’s disease registry data [27]. These findings deserve greater attention to define the exact biological and clinical profiles of both true and incomplete Behçet’s in the UK in case they represent distinct pathobiological entities that are separate from the classic Silk Road descriptions. Our study revealed a low proportion of patients from both Turkish and Middle Eastern ethnic groups, which reflects the demography of Birmingham.

This study has several strengths: This is the first time that the application and diagnostic predictability of the newer ICBD 2014 classification criteria have been investigated for clinical use in a cohort of patients referred to a National Behçet’s Centre in the UK. The patients referred to the service represent an unselected cohort, which arguably assesses a set of discriminatory criteria in a more accurate and real-time manner than selecting out a control group beforehand with other final diagnoses. Our data compare the classification criteria against the conventional clinical diagnosis, and depicts the rate of reclassification in patients who presented with clinical manifestations but did not fulfil the previous classification criteria. This study goes further in defining a relatively under-recognised group of ‘incomplete’ patients who appear to have an undifferentiated inflammatory condition but who do not currently exhibit sufficient diagnostic certainty for BD. Future research may help to further understand the biological factors that are relevant for these patients.

Our study has some limitations. Firstly, it was retrospective and conducted at a single time point, implying that it is difficult to be certain about future development of clinically defining features or severity of disease over time. Secondly, the ophthalmic indicators of BD may have been over-represented in our cohort as our hospital is an internationally renowned tertiary referral centre for uveitis.

## Conclusions

In summary, the in-house data collection system linked to the electronic medical records enabled effective evaluation and investigation of the Birmingham National Behçet’s Centre of Excellence to be undertaken. As expected, the proposed ICBD 2014 criteria were more sensitive at picking up cases than ISG 1990 using the multidisciplinary clinical assessment process as the gold standard. Specificity was less than expected for both criteria but particularly so for ICBD, as in our hands certain clinical features were not always judged to be attributable to a BD diagnosis and gave rise to high false positive values, though time and future follow-ups are likely to improve performance. ICBD may serve as a useful validated screening tool for BD but in our hands in a predominantly UK population, appears to over-diagnose BD. The gold standard for diagnosis should remain the multidisciplinary clinical assessment, and if criteria are to be used to assist with diagnosis, then we would suggest reverting back to the older ISG 1990 criteria until a more suitable alternative can be validated for use in a UK population. This study also highlights the need for further international harmonisation on potential geographic variations in BD clinical presentations. Future research may wish to investigate the revised ICBD criteria in all three UK National Behçet’s Centres.

## Abbreviations

BD: Behçet’s disease
ICBD: International Criteria for Behçet’s disease
ISG: International Study Group

## References

[1] Morton LT, Situnayake D, Wallace GR (2016). Genetics of Behcet’s disease. Curr Opin Rheumatol.

[2] Feigenbaum A (1956). Description of Behçet’s syndrome in the Hippocratic third book of endemic diseases. Br J Opthalmol.

[3] Behçet H. Uber rezidivierende, aphthose durch ein virus verursachte geschwure am mund, am auge und an der genitalen. Dermatol Wochenschr. 1937;1152.

[4] Mutlu S, Scully C (1994). The person behind the eponym: Hulûsi Behçet (1889–1948). J Oral Pathol Med.

[5] Yazici H, Fresko I, Yurdakul S (2007). Behçet’s syndrome: disease manifestations, management, and advances in treatment. Nat Clin Pract Rheumatol.

[6] Yurdakul S, Hamuryudan V, Yazici H (2004). Behcet’s Syndrome. Curr Opin Rheumatol.

[7] Calamia KT, Wilson FC, Icen M, Crowson CS, Gabriel SE, Kremers HM (2009). Epidemiology and clinical characteristics of Behcet’s disease in the US: a population-based study. Arthritis Rheum-Arthritis Care Res.

[8] Dundar SV (1991). Familial clusters of Behcet’s disease. Behcets Dis: Basic Clin Asp.

[9] Akpolat T, Koç Y, Yeniay I, Akpek G, GüllüI I, Kansu E, Kiraz S, Ersoy F, Batman F, Kansu T (1992). Familial Behçet’s disease. Eur J Med.

[10] International Study group for Behçet’s disease (1990). Criteria for the diagnosis of Behçet’s disease. Lancet.

[11] O’Neill T, Rigby A, Silman A, Barnes C (1994). Validation of the International Study Group criteria for Behcet’s disease. Br J Rheumatol.

[12] Davatchi F, Assaad-Khalil S, Calamia KT, Crook JE, Sadeghi-Abdollahi B, Schirmer M, Tzellos T, Zouboulis CC, Akhlagi M, Al-Dalaan A (2014). The International Criteria for Behcet’s Disease (ICBD): a collaborative study of 27 countries on the sensitivity and specificity of the new criteria. J Eur Acad Dermatol Venereol.

[13] Whallett AJ, Thurairajan G, Hamburger J, Palmer RG, Murray PI (1999). Behcet’s syndrome: a multidisciplinary approach to clinical care. QJM-Mon J Assoc Physicians.

[14] Torres R, Yáñez B, Herreras J, Calonge M (2004). Ocular Behçet disease. Retrospective study. Arch Soc Esp Oftamol.

[15] Okada A, Stanford M, Tabbara K (2012). Ancillary testing, diagnostic/classification criteria and severity grading in Behçet disease. Ocul Immunol Inflamm.

[16] June RR, Aggarwal R (2014). The use and abuse of diagnostic/classification criteria. Best Pract Res Clin Rheumatol.

[17] Ohno S, Ohguchi M, Hirose S, Matsuda H, Wakisaka A, Aizawa M (1982). Close association of HLA-Bw51 with Behcet’s disease. Arch Ophthalmol.

[18] de Menthon M, LaValley MP, Maldini C, Guillevin L, Mahr A (2009). HLA-B51/B5 and the risk of Behcet’s disease: a systematic review and meta-analysis of case-control genetic association studies. Arthritis Care Res.

[19] Verity DH, Marr JE, Ohno S, Wallace GR, Stanford MR (1999). Behcet’s disease, the Silk Road and HLA-B51: historical and geographical perspectives. Tissue Antigens.

[20] Arida A, Vaiopoulos G, Markomichelakis N, Kaklamanis P, Sfikakis PP (2009). Are clusters of patients with distinct clinical expression present in Behcet’s disease?. Clin Exp Rheumatol.

[21] Ahmad T, Wallace GR, James T, Neville M, Bunce M, Mulcahy-Hawes K, Armuzzi A, Crawshaw J, Fortune F, Walton R (2003). Mapping the HLA association in Behcet’s disease - A role for tumor necrosis factor polymorphisms?. Arthritis Rheum.

[22] Bennani N, Atouf O, Benseffaj N, Brick C, Essakalli M (2009). HLA polymorphism and Behcet’s disease in Moroccan population. Pathol Biol.

[23] Takeuchi M, Kastner DL, Remmers EF (2015). The immunogenetics of Behcet’s disease: a comprehensive review. J Autoimmun.

[24] Kamiishi T, Itoh Y, Meguro A, Nishida T, Sasaki S, Nanba K, Ohno S, Inoko H, Mizuki N (2008). Four-digit allele genotyping of HLA-A and HLA-B genes in Japanese patients with Behcet’s disease (BD) by a PCR-SSOP-Luminex method and stratification analysis according to each major symptom of BD. Nippon Ganka Gakkai Zasshi.

[25] Meguro A, Inoko H, Ota M, Katsuyama Y, Oka A, Okada E, Yamakawa R, Yuasa T, Fujioka T, Ohno S (2010). Genetics of Behcet disease inside and outside the MHC. Ann Rheum Dis.

[26] Yazici H, Chamberlain MA, Tuzun Y, Yurdakul S, Muftuoglu A (1984). A comparative study of the pathergy reaction among Turkish and British patients with Behçet’s disease. Ann Rheum Dis.

[27] Bonitsis NG, Nguyen LBL, LaValley MP, Papoutsis N, Altenburg A, Koetter I, Micheli C, Maldini C, Mahr A, Zouboulis CC (2015). Gender-specific differences in Adamantiades-Behçet’s disease manifestations: an analysis of the German registry and meta-analysis of data from the literature. Rheumatology.

